# Nationwide analysis of hospital admissions and outcomes of patients with SARS-CoV-2 infection in Austria in 2020 and 2021

**DOI:** 10.1038/s41598-023-35349-4

**Published:** 2023-05-26

**Authors:** Paul Zajic, Michael Hiesmayr, Peter Bauer, David M. Baron, Anastasiia Gruber, Michael Joannidis, Martin Posch, Philipp G. H. Metnitz

**Affiliations:** 1 Division of General Anaesthesiology, Emergency- and Intensive Care Medicine, Medical University of Graz, Auenbruggerplatz 5, 8036 Graz, Austria; 2 Center for Medical Statistics, Informatics and Intelligent Systems, Medical University of Vienna, Vienna, Austria; 3 Department of Anaesthesiology, General Intensive Care Medicine and Pain Medicine, Medical University of Vienna, Vienna, Austria; 4 Division of Intensive Care and Emergency Medicine, Department of Internal Medicine, Medical University of Innsbruck, Innsbruck, Austria

**Keywords:** Epidemiology, Outcomes research, Viral infection, Risk factors, Health services, Prognosis

## Abstract

This retrospective study evaluated temporal and regional trends of patient admissions to hospitals, intensive care units (ICU), and intermediate care units (IMCU) as well as outcomes during the COVID-19 pandemic in Austria. We analysed anonymous data from patients admitted to Austrian hospitals with COVID-19 between January 1st, 2020 and December 31st, 2021. We performed descriptive analyses and logistic regression analyses for in-hospital mortality, IMCU or ICU admission, and in-hospital mortality following ICU admission. 68,193 patients were included, 8304 (12.3%) were primarily admitted to ICU, 3592 (5.3%) to IMCU. Hospital mortality was 17.3%; risk factors were male sex (OR 1.67, 95% CI 1.60–1.75, p < 0.001) and high age (OR 7.86, 95% CI 7.07–8.74, p < 0.001 for 90+ vs. 60–64 years). Mortality was higher in the first half of 2020 (OR 1.15, 95% CI 1.04–1.27, p = 0.01) and the second half of 2021 (OR 1.11, 95% CI 1.05–1.17, p < 0.001) compared to the second half of 2020 and differed regionally. ICU or IMCU admission was most likely between 55 and 74 years, and less likely in younger and older age groups. We find mortality in Austrian COVID-19-patients to be almost linearly associated with age, ICU admission to be less likely in older individuals, and outcomes to differ between regions and over time.

## Introduction

The pandemic spread of SARS-CoV-2 and critical illness entities resulting from coronavirus disease 2019 (COVID-19) such as Acute Respiratory Distress Syndrome (ARDS) and Multi-Organ Dysfunction Syndrome (MODS) have led to significant morbidity and mortality worldwide. First reports from the origin in Wuhan, China have estimated an excess in mortality rates of 56% above the predicted rate^1^.

First studies on infected individuals have reported a spread throughout all age categories^2^. However, patients requiring hospital care have soon been found to be of older age^3^. Similarly, men have been shown to be more likely affected by COVID-19 than women^2^. Thus, it seems reasonable that individuals from different regions, ethnicities, age groups, and sexes may have varying risk to develop severe courses of COVID-19.

The subsequent COVID-19 pandemic has challenged healthcare systems in general and intensive care units (ICUs) in particular all over the world. Reports from various countries have demonstrated differences in both admission policies to ICUs and outcomes from intensive care utilisation in patients suffering from COVID-19^4–7^. Some healthcare systems have experienced states of overload due to caseloads exceeding available resources during the COVID-19 pandemic^8^.

European countries have healthcare systems that differ notably among each other, especially with regards to ICU capacity and provision^9,10^. It stands to reason that these differences in capacities as well as differences in caseloads between countries are associated with variation in resource utilisation and admission policies^11^.

As COVID-19 cases are unevenly distributed regionally, differences in hospital and ICU occupation have been reported. Especially at the beginning of SARS-CoV-2’s spread throughout Europe, regions in the north of Italy have been disproportionally affected^12,13^. It can therefore be suspected that even within single countries, differences in health care utilisation and outcomes thereof occur^14^.

Besides regional differences, the COVID-19 pandemic has also demonstrated a non-linear temporal course of events worldwide. Virus spread and mutation, political decision making, and research efforts into prevention and treatment of the disease have led to phases of higher infection rates and healthcare utilisation, usually referred to as waves^15–18^. It may be hypothesised that intensive care utilisation and outcomes may also vary between these waves.

A possible strategy to increase capacity of facilities that provide care beyond what is usually achievable in normal wards and to improve transition between care levels in hospitals is the utilisation of intermediate care units (IMCU). The value of IMCUs has been demonstrated before COVID-19^19^, and a benefit of a targeted utilisation of these units has been postulated during the pandemic^20^: the use of advanced monitoring and respiratory support seem advantageous, especially in times of limited resources. Conversely, the inability to provide invasive ventilation or extracorporeal life support poses a potential limitation.

### Aim of this study

This study seeks to describe temporal and regional trends of patient admissions to hospitals, ICUs, and IMCUs over the course of the COVID-19 pandemic from 2020 to 2021 in Austria. It aims to elucidate, whether there are differences in outcomes between sexes, age groups, pandemic waves, and Austrian geographic regions and seeks to investigate, whether there are differences in admission rates to intensive care and intermediate care units between these groups. This information is meant to inform the public, decision makers, and health care professionals alike for future planning and preparation.

In the following, we describe methods employed (study design and setting, patient and public involvement, ethical approval and consent to participation, patient population, intensive and intermediate care units, primary and secondary endpoints, measurements and data handling, statistical analysis), we depict results found (patient population, in-hospital mortality, intensive care and intermediate care admission), and we discuss our findings (including strengths and limitations).

## Methods

### Study design and setting

This study was a retrospective analysis of data collected according to national legislation by Austrian hospitals and compiled by the Austrian National Public Health Institute (Gesundheit Österreich GmbH, GÖG). Anonymous data were provided to the study group by GÖG following approval of an open public data request directed to https://datenplattform-covid.goeg.at/.

The data set was originally documented for purposes of quality assurance and reimbursement. Collected information include basic sociodemographic data (age, sex), administrative data (length of ICU, IMCU, and hospital stay), and outcome information (survival status at ICU and hospital discharge) in all patients admitted to Austrian hospitals. In addition, information on severity of illness at ICU admission (measured by Simplified Acute Physiology Score 3 (SAPS 3)^21,22^), and intensity of intensive care provided per day of ICU stay (measured by Simplified Therapeutic Intervention Scoring System (TISS-28)^23^) were provided in patients admitted to ICUs.

### Patient and public involvement

The study was planned and conducted as a collaboration of researchers from all public medical universities in Austria. It sought to inform both the public, officials, and the scientific community about real-world implications of health care provision and effects of pandemics. It was conducted in public data thankfully provided by the Austrian National Public Health Institute and was meant to be distributed throughout the public by means of open access publication.

### Ethical approval and consent to participation

The anonymous fashion of the dataset precluded the need for ethical approval; informed consent was thus neither mandatory nor possible. All used methods were carried out in accordance with relevant guidelines and regulations. The General Data Protection Regulation (GDPR) was not applicable due to the anonymous character of the dataset.

### Patient population

Data on all patient admissions to Austrian acute care hospitals with ICD-10 diagnoses U04.9, U07.1, and U07.2 between January 1st, 2020 and December 31st, 2021 were included in the original dataset. For the conduction of this study, only data on the chronologically first COVID-19-related hospital admission per patient, i.e., any admission due to the aforementioned diagnoses and consecutive transfers and re-admissions, were used. Datasets with missing identifiers were excluded from analyses.

### Intensive and intermediate care units

ICUs and IMCUs were identified within the original dataset according to the definition in the Austrian health structure Plan (Österreichischer Strukturplan Gesundheit, ÖSG): ICUs were units that provide care for patients who require “monitoring and restoration of vital functions, that are deranged in a life-threatening manner and need to be restored or upkept by specific intensive interventions”, IMCUs (including Respiratory Care Units (RCU) and Cardiac Care Units (CCU)) were units that allow for the “monitoring and treatment of patients, whose vital functions are at risk” and “provide the possibility of short-term (i.e., limited to a few days) intensive care (especially invasive mechanical ventilation: 48 h maximum).

### Primary and secondary endpoints

The primary endpoint was in-hospital mortality. Secondary endpoints of interest were primary admission to ICU or IMCU, whichever occurred first. Parameters of interest with possible influence on these endpoints were patient age, sex, region of care, and temporal course of the pandemic (i.e., waves).

### Measurements and data handling

Patient age at hospital admission was reported in categories of five years each in the original dataset to ensure anonymity. Age categories between 0 and 39 years were further condensed into two categories (0–19 years, 20–39 years, respectively) due to a low number of cases in these categories.

Information on the regional area of (primary) hospital admission was provided by assigning every dataset to one of 32 care regions prespecified in the Austrian health structure plan (Österreichischer Strukturplan Gesundheit, ÖSG). Based on this information, care regions were broadly grouped into four regions: North (Salzburg, Upper Austria), East (Vienna, Lower Austria, Burgenland), South (Styria, Carinthia), and West (Tyrol, Vorarlberg).

Information on hospital admission dates were provided in calendar weeks only in order to ensure anonymity of the dataset. To model the time course of the pandemic, half-years were defined from January 1st, 2020 to June 22nd, 2020, June 23rd, 2020 to January 1st, 2021, January 2nd, 2021 to June 21st, 2021, and June 22nd, 2021 to January 1st, 2022, respectively. Information on length of stay was available in days.

Numerical values of SAPS 3 were calculated according to the original publications^21,22^. Data were then categorised in quintiles to circumvent possible deficiencies in adjustment without the need for special customisation^24^ and to allow for the inclusion of datasets with missing SAPS 3 values.

### Statistical analysis

Data description was performed using frequencies and percentages (%) or median and inter-quartile range (IQR), as appropriate.

For the endpoints of in-hospital mortality, admission to IMCU or ICU, and in-hospital mortality following ICU admission, we used a hierarchical logistic regression model with logit link function and fixed factors sex, age and half-year. Additionally, region was included as a fixed factor and care region within region as a random factor (assumed to be conditionally independent) to account for spatial correlation at different levels. Hospital mortality was analysed in the total population, in patients admitted to ICU not previously admitted to IMCU (i.e., primarily admitted to ICU), and in patients admitted to IMCU not previously admitted to an ICU (i.e., primarily admitted to IMCU). For analyses concerning in-hospital mortality following ICU admission adjusted for baseline mortality risk according to the SAPS3 score, the age category of 0–19 years was excluded because the SAPS 3 score was not developed for patients under the age of 18 years^21,22^. Model effects were presented as odds ratios (OR) with 95% confidence intervals (95% CI) compared to the respective reference categories.

For sensitivity analyses, an interaction term between age and sex was included into the multivariable logistic regression analysis models for all endpoints except for the ICU mortality model adjusted for SAPS 3. Further sensitivity analyses encompassed analysis for in-hospital mortality including data with missing identifiers and analyses for in-hospital mortality in all patients who were admitted to ICU or IMCU at any timepoint during their respective hospital stays.

All calculations were performed using R version 4.2.0 with packages lme4 and lmerTest.

### Ethics approval

The anonymous fashion of the dataset precluded the need for ethical approval.

## Results

### Patient population

A total of 68,193 first COVID-19-related hospital admissions were documented in the timeframe of observation (Fig. S1). Of these, 32,319 (47.4%) were female and 35,874 (52.6%) were male. Patients were most commonly of advanced age (Table 1). Most patients were cared for in the Eastern region of Austria (n = 27,952, 41.0%), while fewest were treated in the Western region of Austria (n = 7831, 11.5%). Overall, 11,770 (17.3%) patients died during their respective first COVID-19-related hospital stay. Median length (1^st^–3rd quartile) of hospital stay was 9 (5–17) days.

**Table 1:** Demographics, regional variation, and outcomes of patients admitted to hospital (upper half) and patients admitted to intensive care units (lower half) overall and per half-years.

	Variable	Overall	2020		2021	
			Half-year 1	Half-year 2	Half-year 3	Half-year 4
Patients Admitted to Hospital	*n* of patients	68,193	3191	27,409	20,066	17,527
	Sex (n, %)					
	Female	32,319 (47.4%)	1432 (44.9%)	13,004 (47.4%)	9462 (47.2%)	8421 (48.0%)
	Male	35,874 (52.6%)	1759 (55.1%)	14,405 (52.6%)	10,604 (52.8%)	9106 (52.0%)
	Age category [years] (n, %)					
	up to 19	1774 (2.6%)	40 (1.3%)	511 (1.9%)	516 (2.6%)	707 (4.0%)
	20–39	5361 (7.9%)	216 (6.8%)	1594 (5.8%)	1667 (8.3%)	1884 (10.7%)
	40–44	2041 (3.0%)	81 (2.5%)	593 (2.2%)	672 (3.3%)	695 (4.0%)
	45–49	2975 (4.4%)	151 (4.7%)	879 (3.2%)	1030 (5.1%)	915 (5.2%)
	50–54	4364 (6.4%)	210 (6.6%)	1471 (5.4%)	1466 (7.3%)	1217 (6.9%)
	55–59	5355 (7.9%)	215 (6.7%)	1900 (6.9%)	1803 (9.0%)	1437 (8.2%)
	60–64	5545 (8.1%)	262 (8.2%)	2040 (7.4%)	1812 (9.0%)	1431 (8.2%)
	65–69	5811 (8.5%)	276 (8.6%)	2325 (8.5%)	1781 (8.9%)	1429 (8.2%)
	70–74	7048 (10.3%)	334 (10.5%)	2975 (10.9%)	2077 (10.4%)	1662 (9.5%)
	75–79	8065 (11.8%)	433 (13.6%)	3643 (13.3%)	2223 (11.1%)	1766 (10.1%)
	80–84	9003 (13.2%)	430 (13.5%)	4225 (15.4%)	2286 (11.4%)	2062 (11.8%)
	85–89	6309 (9.3%)	294 (9.2%)	3094 (11.3%)	1533 (7.6%)	1388 (7.9%)
	90 and above	4542 (6.7%)	249 (7.8%)	2159 (7.9%)	1200 (6.0%)	934 (5.3%)
	Region (n, %)					
	East	27,952 (41.0%)	1429 (44.8%)	9923 (36.2%)	10,038 (50.0%)	6562 (37.4%)
	North	18,021 (26.4%)	595 (18.6%)	7905 (28.8%)	4092 (20.4%)	5429 (31.0%)
	South	14,389 (21.1%)	596 (18.7%)	6279 (22.9%)	4009 (20.0%)	3505 (20.0%)
	West	7831 (11.5%)	571 (17.9%)	3302 (12.0%)	1927 (9.6%)	2031 (11.6%)
	In-hospital mortality (n, %)	11,770 (17.3%)	639 (20.0%)	5145 (18.8%)	3177 (15.8%)	2809 (16.0%)
Patients Admitted to Intensive Care Units	*n* of patients	8304	468	2943	2643	2250
	Sex (n, %)					
	Female	2919 (35.2%)	154 (32.9%)	1045 (35.5%)	943 (35.7%)	777 (34.5%)
	Male	5385 (64.8%)	314 (67.1%)	1898 (64.5%)	1700 (64.3%)	1473 (65.5%)
	Age category [years] (n, %)					
	up to 19	115 (1.4%)	4 (0.9%)	33 (1.1%)	34 (1.3%)	44 (2.0%)
	20–39	410 (4.9%)	26 (5.6%)	124 (4.2%)	154 (5.8%)	106 (4.7%)
	40–44	233 (2.8%)	11 (2.4%)	85 (2.9%)	76 (2.9%)	61 (2.7%)
	45–49	368 (4.4%)	23 (4.9%)	121 (4.1%)	122 (4.6%)	102 (4.5%)
	50–54	627 (7.6%)	33 (7.1%)	239 (8.1%)	189 (7.2%)	166 (7.4%)
	55–59	878 (10.6%)	46 (9.8%)	284 (9.7%)	303 (11.5%)	245 (10.9%)
	60–64	973 (11.7%)	55 (11.8%)	359 (12.2%)	288 (10.9%)	271 (12.0%)
	65–69	1116 (13.4%)	68 (14.5%)	392 (13.3%)	346 (13.1%)	310 (13.8%)
	70–74	1238 (14.9%)	75 (16.0%)	435 (14.8%)	405 (15.3%)	323 (14.4%)
	75–79	1078 (13.0%)	67 (14.3%)	404 (13.7%)	330 (12.5%)	277 (12.3%)
	80–84	893 (10.8%)	38 (8.1%)	343 (11.7%)	269 (10.2%)	243 (10.8%)
	85–89	288 (3.5%)	18 (3.8%)	94 (3.2%)	98 (3.7%)	78 (3.5%)
	90 and above	87 (1.0%)	4 (0.9%)	30 (1.0%)	29 (1.1%)	24 (1.1%)
	Region (n, %)					
	East	2899 (34.9%)	173 (37.0%)	954 (32.4%)	999 (37.8%)	773 (34.4%)
	North	2235 (26.9%)	126 (26.9%)	852 (29.0%)	653 (24.7%)	604 (26.8%)
	South	2078 (25.0%)	112 (23.9%)	759 (25.8%)	641 (24.3%)	566 (25.2%)
	West	1092 (13.2%)	57 (12.2%)	378 (12.8%)	350 (13.2%)	307 (13.6%)
	SAPS 3 (median, IQR)	52 (45–60)	51 (45–60)	52 (45–60)	52 (45–60)	52 (45–61)
	In-hospital mortality (n, %)	3047 (36.7%)	180 (38.5%)	1052 (35.7%)	992 (37.5%)	823 (36.6%)

Of all patients, 8304 (12.3%) were primarily admitted to ICU during their respective hospital stays; of these, only 2919 (35.2%) were female and 5385 (64.8%) were male. The age distribution of patients admitted to ICU was different from that of all patients admitted to hospital (Table 1). Regional distribution of care differed somewhat from the overall population (Table 1). 3047 (36.7%) ICU patients died in the hospital; median length of hospital stay was 21 (12–35) days.

Of all patients, 3592 (5.3%) were primarily admitted to IMCU during their respective hospital stays; similar to patients admitted to ICU, only 1316 (37.3%) were female and 2213 (62.7%) were male. Most patients were also of advanced age (Table S1). Regional usage of IMCUs varied considerably (Table S1). 1272 (36.0%) IMCU patients died in the hospital; median length of hospital stay was 18 (11–32) days (Table S1). 942 IMCU patients were later transferred to an ICU; 471 (50.0%) of which died in the hospital.

### In-hospital mortality

**Figure 1:**
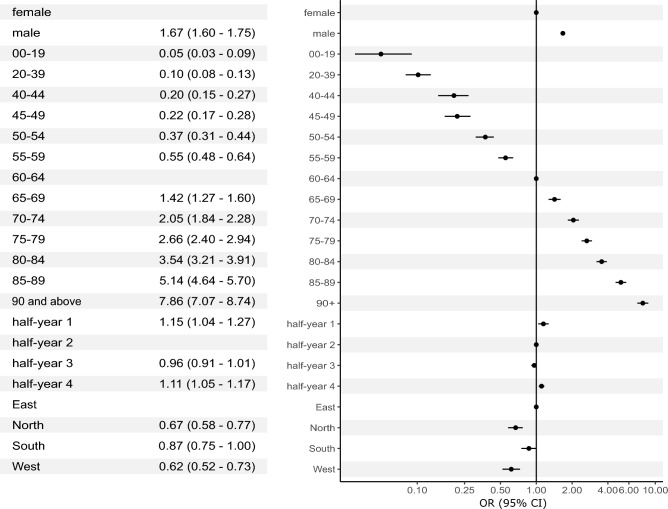
Multivariable logistic regression analysis in all patients with COVID-19 admitted to Austrian hospitals in 2020 and 2021 (n = 68,193) with in-hospital mortality (n = 11,770) as the dependent variable. CI, confidence interval; OR, odds ratio.

In all patients, multivariable logistic regression analysis identified male sex as a risk factor for in-hospital mortality (OR 1.67, 95% CI 1.60–1.75, p < 0.001). There was an almost linear association between age and risk of in-hospital mortality. Compared to the reference group (60–64 years), patients up to 19 years of age had lowest odds (OR 0.05, 95% CI 0.03–0.09, p < 0.001) and patients aged 90 years or above had highest odds to die in the hospital (OR 7.86, 95% CI 7.07–8.74, p < 0.001) (Fig. 1).

Some variation in outcomes over time was notable: compared to half-year 2, risk of in-hospital mortality was higher in half-year 1 (OR 1.15, 95% CI 1.04–1.27, p = 0.01) and half-year 4 (OR 1.11, 95% CI 1.05–1.17, p < 0.001) (see Fig. S2 for comparison of unadjusted in-hospital mortalities). There was also significant geographical variation in outcomes: risk of in-hospital mortality was lower in all other regions compared to the Eastern region of Austria (Fig. 1).

Sensitivity analyses did not detect significant interactions between patient age and sex (Table S2). Findings were virtually identical when datasets with missing patient identifiers were included (Table S3).

**Figure 2:**
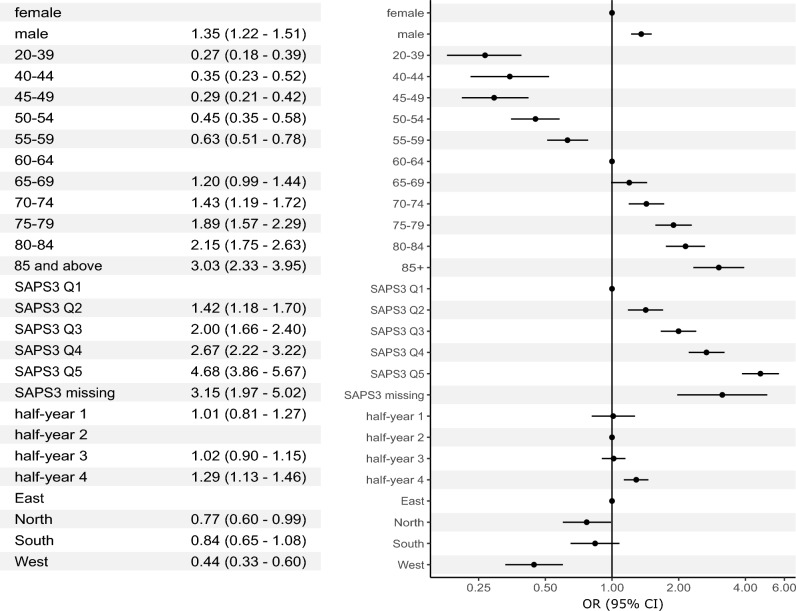
Multivariable logistic regression analysis in patients with COVID-19 admitted to Austrian intensive care units in 2020 and 2021 (*n* = 8304) with in-hospital mortality (*n* = 3047) as the dependent variable. CI, confidence interval; OR, odds ratio.

In patients primarily admitted to ICUs, analysis adjusted for baseline risk of mortality according to the SAPS 3 score yielded similar results. Men were at increased risk of in-hospital mortality (OR 1.35, 95% CI 1.22–1.51, p < 0.001) (Fig. 2). The quasi-linear association between age category and outcomes was also present in this patient group; compared to 60–64 years, lowest odds for mortality were found in patients aged 20–39 years (OR 0.27, 95% CI 0.18–0.39, p < 0.001), highest odds were found in those aged 85 years and above (OR 3.03, 95% CI 2.33–3.95, p < 0.001) (Fig. 2, Fig. S4).

There was little variation in outcomes over time in the IMCU patient group: mortality was virtually identical in half-years 1, 2, and 3, but was significantly higher in half-year 4 (OR 1.29, 95% CI 1.13–1.46, p < 0.001). Regional variation was also evident; highest risk of mortality was found in the reference category of Eastern Austria, whereas lowest odds were found in the West (OR 0.44, 95% CI 0.33–0.60, p < 0.001) (Table S4, Fig. S5).

### Intensive and intermediate care admission

**Figure 3:**
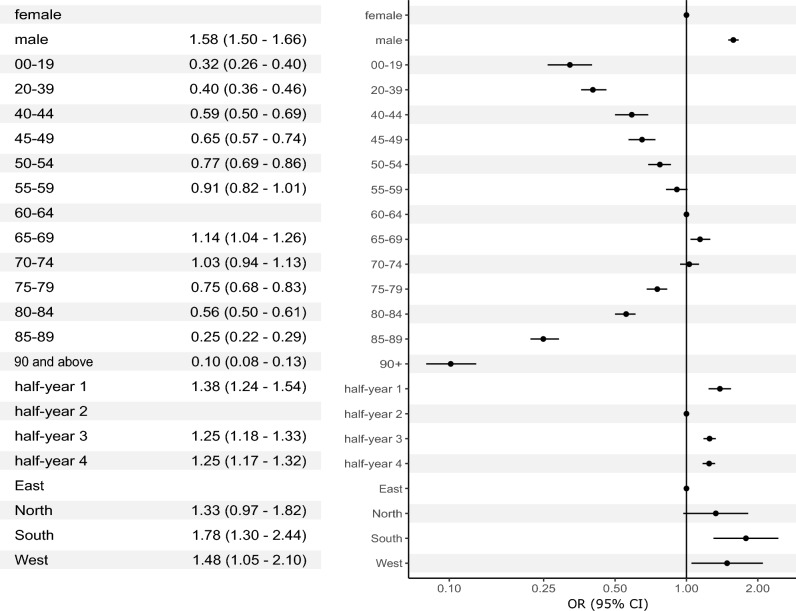
Multivariable logistic regression analysis in all patients with COVID-19 admitted to Austrian hospitals in 2020 and 2021 (n = 68,193) with primary admission to an intensive care unit (n = 8304) as the dependent variable. CI, confidence interval; OR, odds ratio.

In multivariable logistic regression analysis, men were found to be significantly more likely to be admitted to ICU than women (OR 1.58, 95% CI 1.50–1.66, p < 0.001). Patients aged 55–74 years were most likely to be admitted to ICU, whereas the odds were drastically lower in both younger and older patients (Fig. 3).

The chance of ICU admission varied over time; lowest odds were observed in the second half-year, when case-load was highest. Regional differences could also be discerned; patients were least likely to be admitted to an ICU in the Eastern region of Austria (were most COVID-19 cases were registered), whereas the chance was highest in the South of Austria (OR 1.78, 95% CI 1.30–2.44, p < 0.001 compared to East) (Fig. 3).

Similarly, admission to an IMCU was less likely in patients of younger and older age; however, significantly lower odds in older individuals were found in the age groups from 80 years and above (Table S4). Patients were predominantly male as well (OR 1.46, 95% CI 1.36–1.57, p < 0.001). Regional differences in IMCU utilisation were striking; odds of IMCU admission were lower in all other regions than in the Eastern region of Austria (Table S5).

Results for both ICU and IMCU admission were not altered drastically by the inclusion of an interaction term between age category and patient sex (Tables S6, S7).

**Figure 4:**
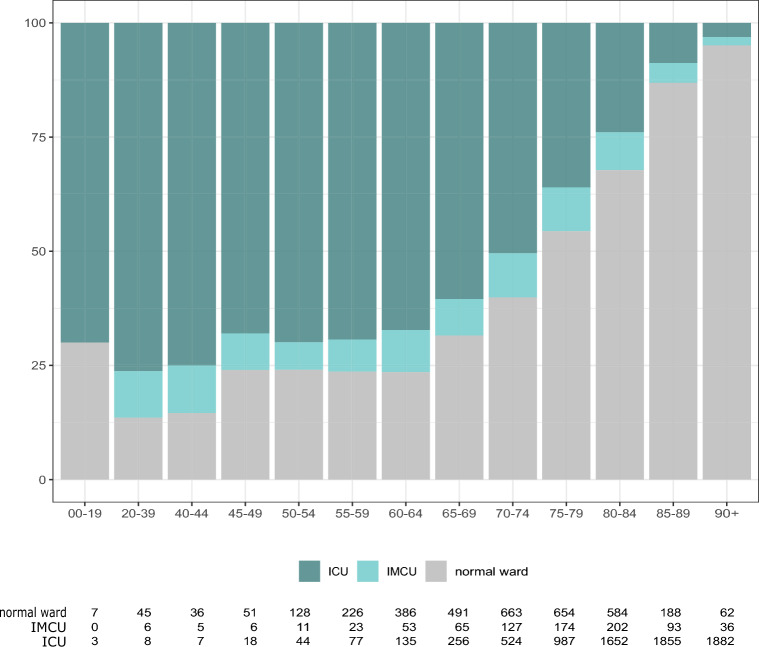
Proportions of highest care facility to which patients who died in hospital (n = 11,770) had been admitted, stratified by age. x-axis, age categories; y-axis, admission to highest care facility; ICU, admission to Intensive Care Unit (ICU); IMCU, admission to Intermediate Care Units (IMCU); normal ward, admission to neither (treated in normal wards only).

Descriptive analysis of patients who died in hospital underlines the relatively restrictive allocation of ICU and IMCU capacities to individuals of older age. While in the age groups up to 64 years, fewer than 25% of all patients, who would decease during their hospital stay, were never admitted to ICU or IMCU, this proportion increased steadily with every following age category (Fig. 4).

## Discussion

In this large study conducted in data on all patients admitted to Austrian hospitals between 2020 and 2021 with a documented SARS-CoV-2 infection, we describe patient demographics, short-term mortality, and resource use. Furthermore, we explore regional differences and changes over time.

Our findings underline the notion that men are at increased risk of severe COVID-19. While male individuals made up 52.6% of all patients admitted to hospital with SARS-CoV-2, which is not drastically different to the proportion of men in the Austrian population (49.2%^25^), they accounted for almost two thirds of all patients admitted to ICU. Regression analyses identified male sex as a risk factor for ICU admission and in-hospital mortality, which is in accordance with findings from other studies^26^.

We find approximately 60% of patients admitted to hospital with SARS-CoV-2 infections to be 65 years or older in age. In comparison, out of an average population count of 8,916,845 in 2020, only 1,707,773 (19.2%) individuals belong to that age group^25^. This is in line with previous findings that age is directly correlated with the risk for hospital admission due to COVID-19^27^. Moreover, we find risk of short-term mortality in all patients hospitalised with SARS-CoV-2 to be drastically higher in older patients than in younger patients. This, again, is in accordance with previous studies^27–30^. Of note, this effect persists in multivariable analysis although SAPS 3 already adjusts for age^21,22^.

We also find younger individuals (especially those below the age of 40) and older individuals (especially those aged 80 and above) less likely to be admitted to ICU for treatment. While this may be explained by a lower mortality risk in younger patients, a biological explanation for this finding seems less plausible in older patients. Similar admission characteristics with regards to patient age have been found in other European countries^31^. Guidet et al.^32^ report that withholding and withdrawing of life-sustaining treatments from very old patients is more common in COVID-19 compared to other reasons for acute respiratory failure.

It seems likely that these findings are the result of decision making, either based on patients’ wishes, perceived meaningfulness of treatment, or frank triage due to capacity. Similar observations have been made in a multinational, multicentre cohort study in critically ill elderly COVID-19 patients^33^. Of note, there has been no formal policy allowing or disallowing for age-based patient selection in Austria in the concerned time period.

Although very old patients are also less likely to be treated in IMCUs compared to mid-aged individuals, IMCU admission of older individuals is comparably more likely than ICU admission. Mortality rates, however, are almost identical in patients admitted to ICU and to IMCU. In patients admitted to ICU after an IMCU stay, mortality is very high at 50%.

Findings are unevenly distributed both geographically and temporally. The unadjusted overall patient distribution over the East, North, South, and West regions of 41.0%, 26.4%, 21.1%, and 11.5%, respectively, somewhat resembles the population distribution of 43.6%, 22.9%, 18.0%, and 11.5%^25^. However, this distribution varied notably over time, underscoring the comparably high caseloads in the West in the first half-year and in the East in the third half-year.

Regression analyses demonstrate that the risks of ICU admission and in-hospital mortality have been pronounced in half-year 1 at the beginning of the pandemic and later in half-year 4. Possible explanations encompass regional capacity shortages, changes in treatment over time, vaccination, and virus variants^34,35^. Higher mortality at the end of the observation time frame compared to the beginning underscores the fact, that improvements in treatment and outcomes are non-linear, contrary to earlier findings from other countries^30^.

Analyses highlight notable differences in mortality risks overall and following intensive care between regions. A possible explanation lies in different prior resource availability, acute resource utilisation; for instance, the state of Tyrol in the Western region has had structured resource management already established with the beginning of the first wave^11^. Variation in treatments applied for COVID-19 in intensive care units has been found previously^36^; outcomes in relatively small regions may be even more dependent on performance of individual centres. Another possible explanation can be found in differences in baseline life expectancy: men and women have respective average life expectancies of 80.5 and 84.9 years in the Western region of Austria compared to 78.6 and 83.2 years in the East^25^.

Findings from other European whole-nation studies demonstrate both similarities and differences. In Sweden, male sex has also been identified as a risk-factor for hospitalisation and ICU admission; similarly, old age has been found a significant predictor of both hospital admission and ICU non-admission. This effect, however, is present at ages above 69 years in Sweden, while it is only visible in the age groups of 75 years and above in Austria^37^. In the Netherlands, mortality has been found lowest in the so-called third wave, broadly similar to half-year 3 in this study^38^.

### Strengths and limitations

This is a large study conducted using nationwide data. Data completeness is adequate as data collection is mandated by legislation. Data granularity, however, is limited: Information on acute health status and chronic conditions is only available in patients admitted to intensive care units. No biometric data apart from age and sex are available; specific information on treatment and medium-to-long-term outcomes are not part of collected data. Patient age and admission dates have also been deliberately blurred to ensure anonymity of the dataset. Outcome data are limited to in-hospital mortality.

## Conclusion

Patients admitted to Austrian hospitals with SARS-CoV-2 are predominantly of older age. Mortality is almost linearly associated with age. Men are at increased risk of intensive care admission and death. There are significant differences in outcomes between Austrian regions. Admission to intensive care units is drastically less likely in older individuals.

## Supplementary Information

Supplementary Information.

## Data Availability

The data underlying this article will be shared on reasonable request to the corresponding author. Anonymous data from the Austrian National Public Health Institute (Gesundheit Österreich GmbH, GÖG) are principally available upon approval of requests sent in via https://datenplattform-covid.goeg.at/.
