# Supplementary material for: Nationwide analysis of hospital admissions and outcomes of patients with SARS-CoV-2 infection in Austria in 2020 and 2021
Source: Sci Rep. 2023 May 26;13:8548. doi: 10.1038/s41598-023-35349-4 (PMC10214364; doi:10.1038/s41598-023-35349-4)
Supplement: Supplementary file 1 — Supplementary Information. [file 41598_2023_35349_MOESM1_ESM.pdf]

SUPPLEMENTARY MATERIALS

Nationwide analysis of hospital admissions and outcomes of patients with SARS-CoV-2 infection in Austria in 2020 and 2021

Paul Zajic, MD PhD
Division of General Anaesthesiology, Emergency- and Intensive Care Medicine, Medical University of Graz, Graz, Austria

Michael Hiesmayr, MD
Center for Medical Statistics, Informatics and Intelligent Systems, Medical University of Vienna, Vienna, Austria

Peter Bauer, PhD
Center for Medical Statistics, Informatics and Intelligent Systems, Medical University of Vienna, Vienna, Austria

David M. Baron, MD PhD
Department of Anaesthesiology, General Intensive Care Medicine and Pain Medicine, Medical University of Vienna, Vienna, Austria

Anastasiia Gruber, MSc
Center for Medical Statistics, Informatics and Intelligent Systems, Medical University of Vienna, Vienna, Austria

Michael Joannidis, MD
Division of Intensive Care and Emergency Medicine, Department of Internal Medicine, Medical University of Innsbruck, Innsbruck, Austria

Martin Posch, PhD
Center for Medical Statistics, Informatics and Intelligent Systems, Medical University of Vienna, Vienna, Austria

Philipp G. H. Metnitz, MD PhD
Division of General Anaesthesiology, Emergency- and Intensive Care Medicine, Medical University of Graz, Graz, Austria

Table of Contents

Figure S 1 Study Flow Chart........ 3

Table S 1 Demographics, regional variation, and outcomes of patients admitted to hospital (upper half) and patients admitted to intermediate care units (lower half) overall and per half-years. 4

Table S 2 Multivariable logistic regression analysis in all patients with COVID-19 admitted to Austrian hospitals in 2020 and 2021 (*n*=68 193) with in-hospital mortality (*n*=11 770) as the dependent variable including an interaction term between sex and age category. CI = confidence interval, OR = odds ratio. 5

Table S 3 Multivariable logistic regression analysis in all patients with COVID-19 admitted to Austrian hospitals in 2020 and 2021 including those with missing patient identifiers (*n*=73 120) with in-hospital mortality (*n*=12 236) as the dependent variable. CI = confidence interval, OR = odds ratio 6

Figure S 2 Unadjusted in-hospital mortality rates in all patients with COVID-19 admitted to Austrian hospitals in 2020 and 2021 (*n*=68 193) stratified for age categories and separated for half-year of hospital admission (January 1st, 2020 to June 22nd, 2020, June 23rd, 2020 to January 1st, 2021, January 2nd, 2021 to June 21st, 2021, and June 22nd, 2021 to January 1st, 2022) and patient sex. 7

Figure S 3 Boxplot comparison of Simplified Acute Physiology Score 3 (SAPS 3) values between male and female individuals. 8

Figure S 4 Unadjusted in-hospital mortality rates in patients with COVID-19 primarily admitted to intensive care units in 2020 and 2021 (*n*=8 304) stratified for age categories and separated for half-year of hospital admission (January 1st, 2020 to June 22nd, 2020, June 23rd, 2020 to January 1st, 2021, January 2nd, 2021 to June 21st, 2021, and June 22nd, 2021 to January 1st, 2022) and patient sex........ 9

Table S 4 Multivariable logistic regression analysis in patients with COVID-19 admitted to Austrian intermediate care units in 2020 and 2021 (*n*=3 529) with in-hospital mortality (*n*=1 272) as the dependent variable. CI = confidence interval, OR = odds ratio........ 10

Figure S 5 Unadjusted in-hospital mortality rates in patients with COVID-19 primarily admitted to intermediate care units in 2020 and 2021 (*n*=3 529) stratified for age categories and separated for half-year of hospital admission (January 1st, 2020 to June 22nd, 2020, June 23rd, 2020 to January 1st, 2021, January 2nd, 2021 to June 21st, 2021, and June 22nd, 2021 to January 1st, 2022) and patient sex........ 11

Table S 5 Multivariable logistic regression analysis in all patients with COVID-19 admitted to Austrian hospitals in 2020 and 2021 (n=68 193) with primary admission to an intermediate care unit (n=3 529) as the dependent variable. CI = confidence interval, OR = odds ratio. 12

Table S 6 Multivariable logistic regression analysis in all patients with COVID-19 admitted to Austrian hospitals in 2020 and 2021 (*n*=68 193) with primary admission to an intensive care unit (*n*=8 304) as the dependent variable including an interaction term between sex and age category. CI = confidence interval, OR = odds ratio. 13

Table S 7 Multivariable logistic regression analysis in all patients with COVID-19 admitted to Austrian hospitals in 2020 and 2021 (*n*=68 193) with primary admission to an intermediate care unit (*n*=3 529) as the dependent variable including an interaction term between sex and age category. CI = confidence interval, OR = odds ratio. 14

Figure S 1 Study Flow Chart

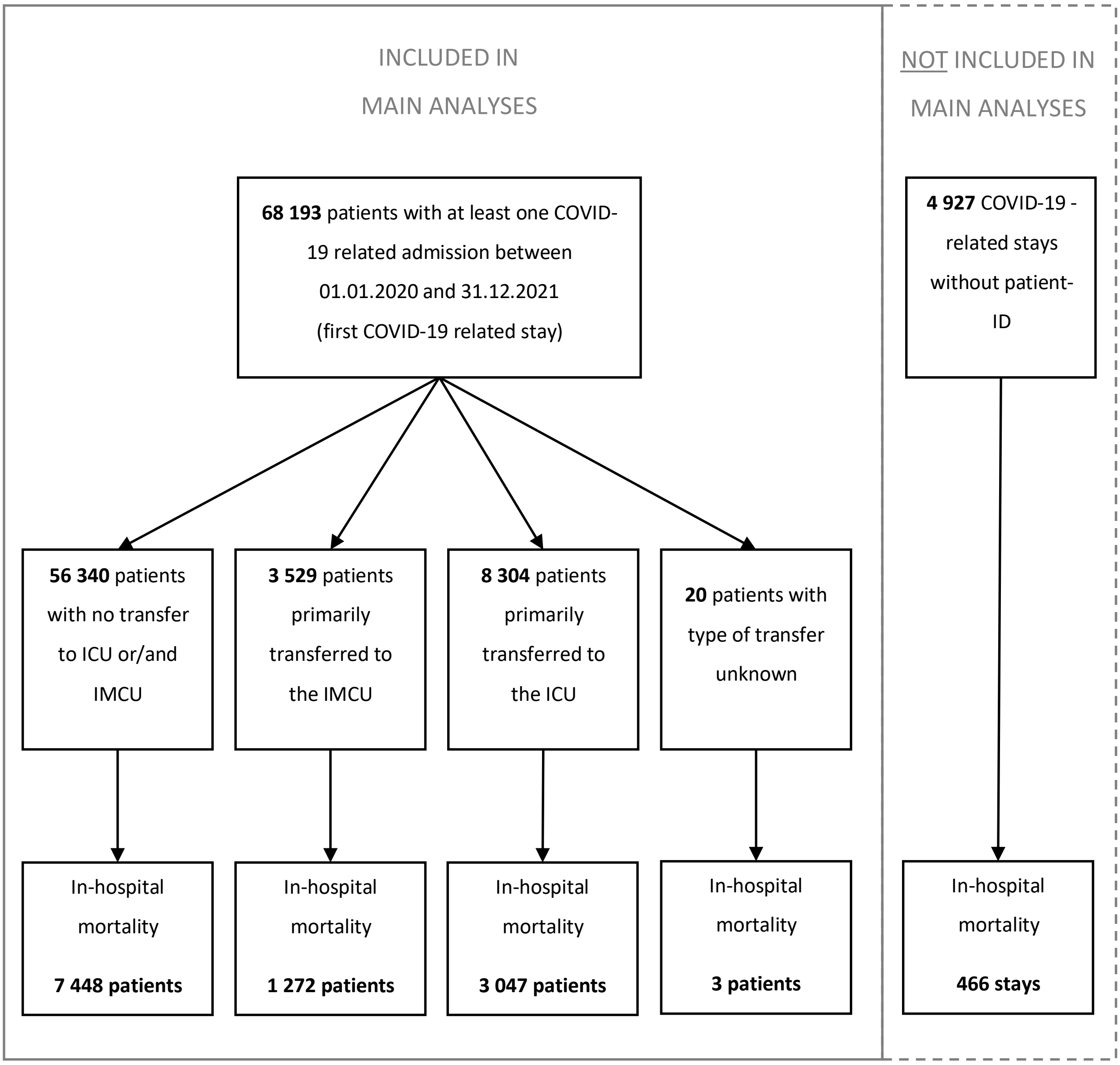

Table S 1 Demographics, regional variation, and outcomes of patients admitted to hospital (upper half) and patients admitted to intermediate care units (lower half) overall and per half-years.

	Variable	Overall	*2020* Half-year 1	*2020* Half-year 2	*2021* Half-year 3	*2021* Half-year 4
**Patients Admitted to Hospital**	*n* of patients	68193	3191	27409	20066	17527
	Sex (n, %)					
	Female	32319 (47.4%)	1432 (44.9%)	13004 (47.4%)	9462 (47.2%)	8421 (48.0%)
	Male	35874 (52.6%)	1759 (55.1%)	14405 (52.6%)	10604 (52.8%)	9106 (52.0%)
	Age category [years] (n, %)					
	up to 19	1774 (2.6%)	40 (1.3%)	511 (1.9%)	516 (2.6%)	707 (4.0%)
	20 – 39	5361 (7.9%)	216 (6.8%)	1594 (5.8%)	1667 (8.3%)	1884 (10.7%)
	40 – 44	2041 (3.0%)	81 (2.5%)	593 (2.2%)	672 (3.3%)	695 (4.0%)
	45 – 49	2975 (4.4%)	151 (4.7%)	879 (3.2%)	1030 (5.1%)	915 (5.2%)
	50 – 54	4364 (6.4%)	210 (6.6%)	1471 (5.4%)	1466 (7.3%)	1217 (6.9%)
	55 – 59	5355 (7.9%)	215 (6.7%)	1900 (6.9%)	1803 (9.0%)	1437 (8.2%)
	60 – 64	5545 (8.1%)	262 (8.2%)	2040 (7.4%)	1812 (9.0%)	1431 (8.2%)
	65 – 69	5811 (8.5%)	276 (8.6%)	2325 (8.5%)	1781 (8.9%)	1429 (8.2%)
	70 – 74	7048 (10.3%)	334 (10.5%)	2975 (10.9%)	2077 (10.4%)	1662 (9.5%)
	75 – 79	8065 (11.8%)	433 (13.6%)	3643 (13.3%)	2223 (11.1%)	1766 (10.1%)
	80 – 84	9003 (13.2%)	430 (13.5%)	4225 (15.4%)	2286 (11.4%)	2062 (11.8%)
	85 – 89	6309 (9.3%9	294 (9.2%)	3094 (11.3%)	1533 (7.6%)	1388 (7.9%)
	90 and above	4542 (6.7%)	249 (7.8%)	2159 (7.9%)	1200 (6.0%)	934 (5.3%)
	Region (n, %)					
	East	27952 (41.0%)	1429 (44.8%)	9923 (36.2%)	10038 (50.0%)	6562 (37.4%)
	North	18021 (26.4%)	595 (18.6%)	7905 (28.8%)	4092 (20.4%)	5429 (31.0%)
	South	14389 (21.1%)	596 (18.7%)	6279 (22.9%)	4009 (20.0%)	3505 (20.0%)
	West	7831 (11.5%)	571 (17.9%)	3302 (12.0%)	1927 (9.6%)	2031 (11.6%)
	In-hospital mortality (n, %)	11770 (17.3%)	639 (20.0%)	5145 (18.8%)	3177 (15.8%)	2809 (16.0%)
**Patients Admitted to Intermediate Care Units**	*n* of patients	3529	159	1396	1255	719
	Sex (n, %)					
	Female	1316 (37.3%)	58 (36.5%)	524 (37.5%)	468 (37.3%)	266 (37.0%)
	Male	2213 (62.7%)	101 (63.5%)	872 (62.5%)	787 (62.7%)	453 (63.0%)
	Age category [years] (n, %)					
	up to 19	45 (1.3%)	0 (0.0%)	10 (0.7%)	18 (1.4%)	17 (2.4%)
	20 – 39	162 (4.6%)	7 (4.4%)	47 (3.4%)	59 (4.7%)	49 (6.8%)
	40 – 44	81 (2.3%)	2 (1.3%)	29 (2.1%)	27 (2.2%)	23 (3.2%)
	45 – 49	139 (3.9%)	4 (2.5%)	39 (2.8%)	54 (4.3%)	42 (5.8%)
	50 – 54	240 (6.8%)	7 (4.4%)	76 (5.4%)	92 (7.3%)	65 (9.0%)
	55 – 59	335 (9.5%)	7 (4.4%)	129 (10.0%)	133 (10.6%)	66 (9.2%)
	60 – 64	376 (10.7%)	17 (10.7%)	140 (10.4%)	141 (11.2%)	78 (10.8%)
	65 – 69	414 (11.7%)	16 (10.1%)	145 (14.4%)	152 (12.1%)	101 (14.0%)
	70 – 74	515 (14.6%)	32 (20.1%)	201 (16.0%)	186 (14.8%)	96 (13.4%)
	75 – 79	511 (14.5%)	35 (22.0%)	223 (16.0%)	183 (14.6%)	70 (9.7%)
	80 – 84	456 (12.9%)	18 (11.3%)	224 (16.0%)	138 (11.0%)	76 (10.6%)
	85 – 89	182 (5.2%)	8 (5.0%)	101 (7.2%)	45 (3.6%)	28 (3.9%)
	90 and above	73 (2.1%)	6 (3.8%)	32 (2.3%)	27 (2.2%)	8 (1.1%)
	Region (n, %)					
	East	2427 (68.8%)	108 (67.9%)	908 (65.0%)	942 (75.1%)	469 (65.2%)
	North	415 (11.8%)	13 (8.2%)	197 (14.1%)	108 (8.6%)	97 (13.5%)
	South	396 (11.2%)	7 (4.4%)	174 (12.5%)	139 (11.1%)	76 (10.6%)
	West	291 (8.2%)	31 (19.5%)	117 (8.4%)	66 (5.3%)	77 (10.7%)
	In-hospital mortality (n, %)	1272 (36.0%)	55 (34.6%)	517 (37.0%)	441 (35.1%)	259 (36.0%)

Table S 2 Multivariable logistic regression analysis in all patients with COVID-19 admitted to Austrian hospitals in 2020 and 2021 (*n*=68 193) with in-hospital mortality (*n*=11 770) as the dependent variable including an interaction term between sex and age category. CI = confidence interval, OR = odds ratio.

Variable	OR	95% CI		*p*
Sex				
Female	1.00			
Male	1.58	1.31	1.91	<0.001
Age category [years]				
up to 19	0.02	0.01	0.09	<0.001
20 – 39	0.08	0.05	0.13	<0.001
40 – 44	0.21	0.13	0.36	<0.001
45 – 49	0.29	0.19	0.42	<0.001
50 – 54	0.37	0.27	0.51	<0.001
55 – 59	0.56	0.43	0.73	<0.001
60 – 64	1.00			
65 – 69	1.43	1.17	1.76	<0.001
70 – 74	1.96	1.62	2.36	<0.001
75 – 79	2.54	2.12	3.04	<0.001
80 – 84	3.25	2.73	3.86	<0.001
85 – 89	4.91	4.13	5.84	<0.001
90 and above	7.71	6.48	9.18	<0.001
Interaction: Sex × Age category				
sex × up to 19	2.64	0.60	11.63	0.20
sex × 20-39	1.38	0.79	2.41	0.26
sex × 40-44	0.93	0.50	1.74	0.82
sex × 45-49	0.65	0.39	1.08	0.10
sex × 50-54	1.00	0.69	1.46	0.99
sex × 55-59	0.97	0.71	1.33	0.87
sex × 60-64	1.00			
sex × 65-69	0.99	0.77	1.26	0.92
sex × 70-74	1.07	0.85	1.34	0.57
sex × 75-79	1.07	0.86	1.33	0.56
sex × 80-84	1.15	0.93	1.43	0.18
sex × 85-89	1.07	0.86	1.33	0.53
sex × 90 and above	1.00	0.80	1.25	0.98
Half-year				
Half-year 1	1.15	1.04	1.27	0.01
Half-year 2	1.00			
Half-year 3	0.96	0.91	1.01	0.16
Half-year 4	1.11	1.05	1.17	<0.001
Region				
East	1.00			
North	0.67	0.58	0.77	<0.001
South	0.87	0.75	1.00	0.05
West	0.62	0.52	0.73	<0.001

Table S 3 Multivariable logistic regression analysis in all patients with COVID-19 admitted to Austrian hospitals in 2020 and 2021 including those with missing patient identifiers (*n*=73 120) with in-hospital mortality (*n*=12 236) as the dependent variable. CI = confidence interval, OR = odds ratio

Variable		OR	95% CI		*p*
Sex					
	Female	1.00			
	Male	1.67	1.60	1.74	<0.001
Age category [years]					
	up to 19	0.05	0.03	0.09	<0.001
	20 – 39	0.10	0.08	0.13	<0.001
	40 – 44	0.20	0.15	0.27	<0.001
	45 – 49	0.23	0.19	0.29	<0.001
	50 – 54	0.38	0.32	0.45	<0.001
	55 – 59	0.56	0.48	0.64	<0.001
	60 – 64	1.00			
	65 – 69	1.44	1.29	1.61	<0.001
	70 – 74	2.05	1.85	2.27	<0.001
	75 – 79	2.65	2.40	2.92	<0.001
	80 – 84	3.58	3.25	3.95	<0.001
	85 – 89	5.18	4.68	5.72	<0.001
	90 and above	7.80	7.03	8.65	<0.001
Half-year					
	Half-year 1	1.11	1.01	1.22	0.04
	Half-year 2	1.00			
	Half-year 3	0.97	0.92	1.02	0.19
	Half-year 4	1.12	1.06	1.18	<0.001
Region					
	East	1.00			
	North	0.66	0.58	0.77	<0.001
	South	0.87	0.76	1.01	0.06
	West	0.60	0.51	0.71	<0.001

Figure S 2 Unadjusted in-hospital mortality rates in all patients with COVID-19 admitted to Austrian hospitals in 2020 and 2021 (*n*=68 193) stratified for age categories and separated for half-year of hospital admission (January 1st, 2020 to June 22nd, 2020, June 23rd, 2020 to January 1st, 2021, January 2nd, 2021 to June 21st, 2021, and June 22nd, 2021 to January 1st, 2022) and patient sex.

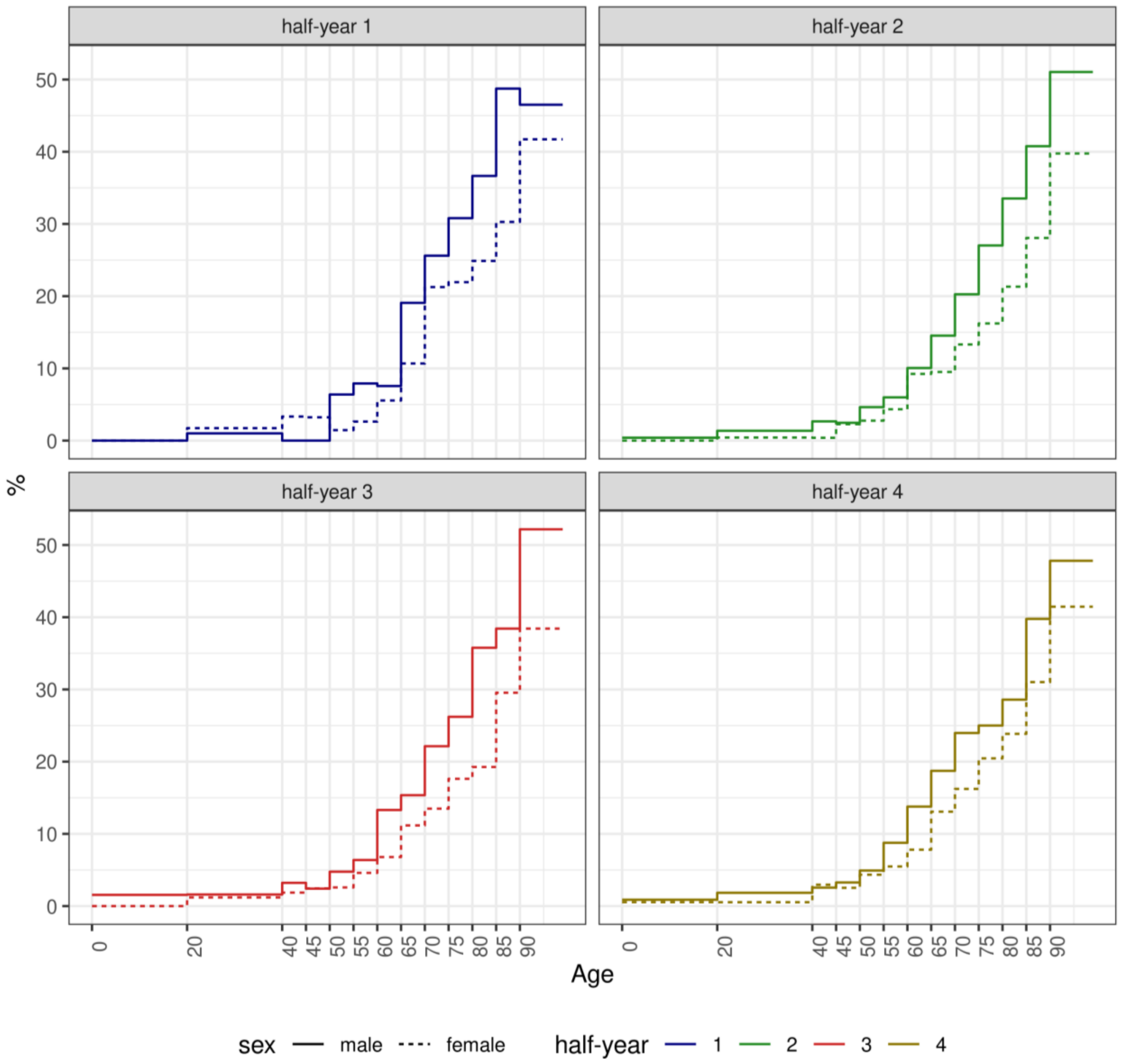

Figure S 3 Boxplot comparison of Simplified Acute Physiology Score 3 (SAPS 3) values between male and female individuals.

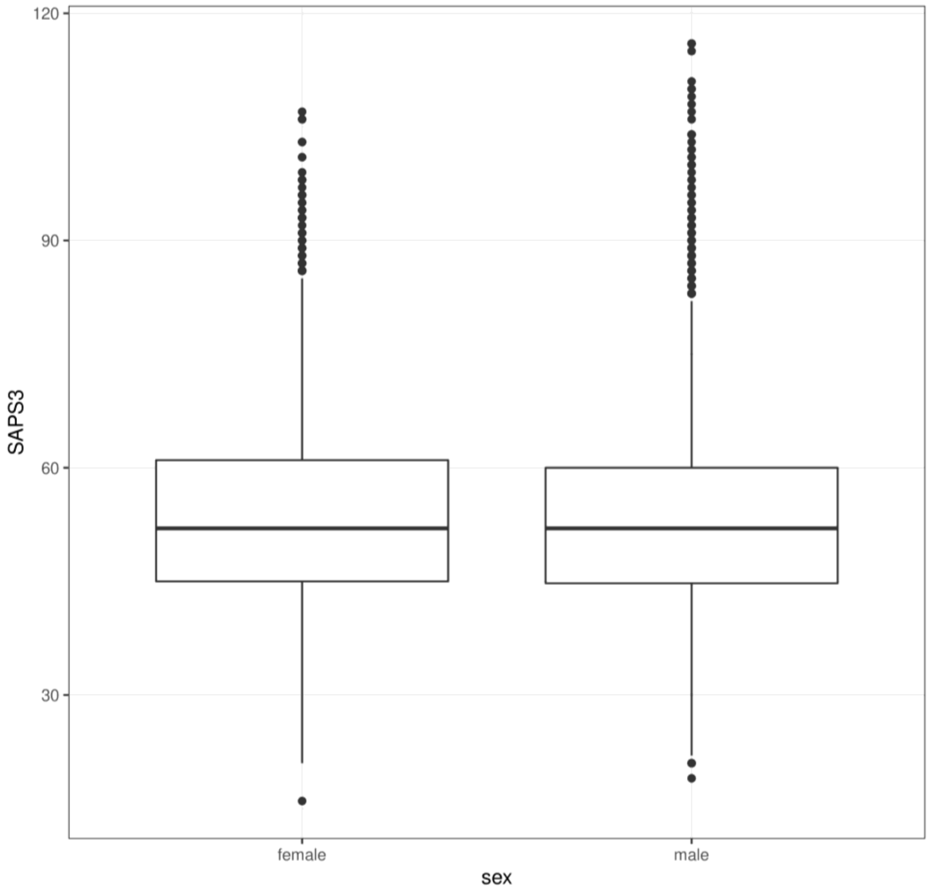

Figure S 4 Unadjusted in-hospital mortality rates in patients with COVID-19 primarily admitted to intensive care units in 2020 and 2021 (*n*=8 304) stratified for age categories and separated for half-year of hospital admission (January 1st, 2020 to June 22nd, 2020, June 23rd, 2020 to January 1st, 2021, January 2nd, 2021 to June 21st, 2021, and June 22nd, 2021 to January 1st, 2022) and patient sex.

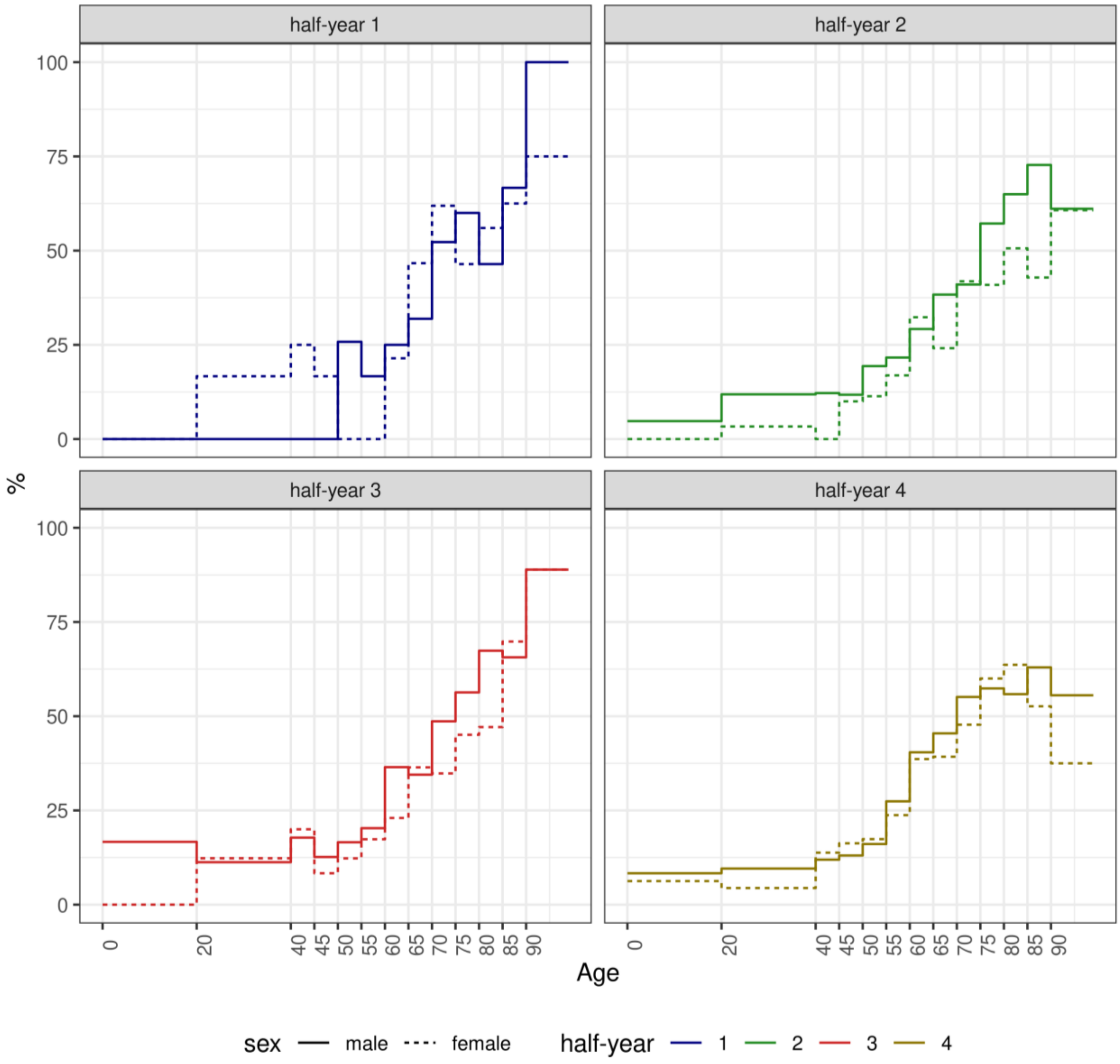

Table S 4 Multivariable logistic regression analysis in patients with COVID-19 admitted to Austrian intermediate care units in 2020 and 2021 (*n*=3 529) with in-hospital mortality (*n*=1 272) as the dependent variable. CI = confidence interval, OR = odds ratio.

Variable		OR	95% CI		*p*
Sex					
	Female	1.00			
	Male	1.53	1.31	1.79	<0.001
Age category [years]					
	20 – 39	0.21	0.12	0.39	<0.001
	40 – 44	0.29	0.14	0.58	<0.001
	45 – 49	0.22	0.12	0.40	<0.001
	50 – 54	0.36	0.24	0.56	<0.001
	55 – 59	0.46	0.32	0.66	<0.001
	60 – 64	1.00			
	65 – 69	1.23	0.91	1.66	0.19
	70 – 74	1.85	1.39	2.45	<0.001
	75 – 79	2.53	1.90	3.37	<0.001
	80 – 84	3.58	2.66	4.81	<0.001
	85 and above	3.41	2.43	4.80	<0.001
Half-year					
	Half-year 1	0.88	0.60	1.27	0.48
	Half-year 2	1.00			
	Half-year 3	1.09	0.91	1.29	0.36
	Half-year 4	1.33	1.08	1.63	0.01
Region					
	East	1.00			
	North	0.82	0.58	1.18	0.29
	South	0.80	0.56	1.15	0.23
	West	0.88	0.59	1.30	0.51

Figure S 5 Unadjusted in-hospital mortality rates in patients with COVID-19 primarily admitted to intermediate care units in 2020 and 2021 (*n*=3 529) stratified for age categories and separated for half-year of hospital admission (January 1st, 2020 to June 22nd, 2020, June 23rd, 2020 to January 1st, 2021, January 2nd, 2021 to June 21st, 2021, and June 22nd, 2021 to January 1st, 2022) and patient sex.

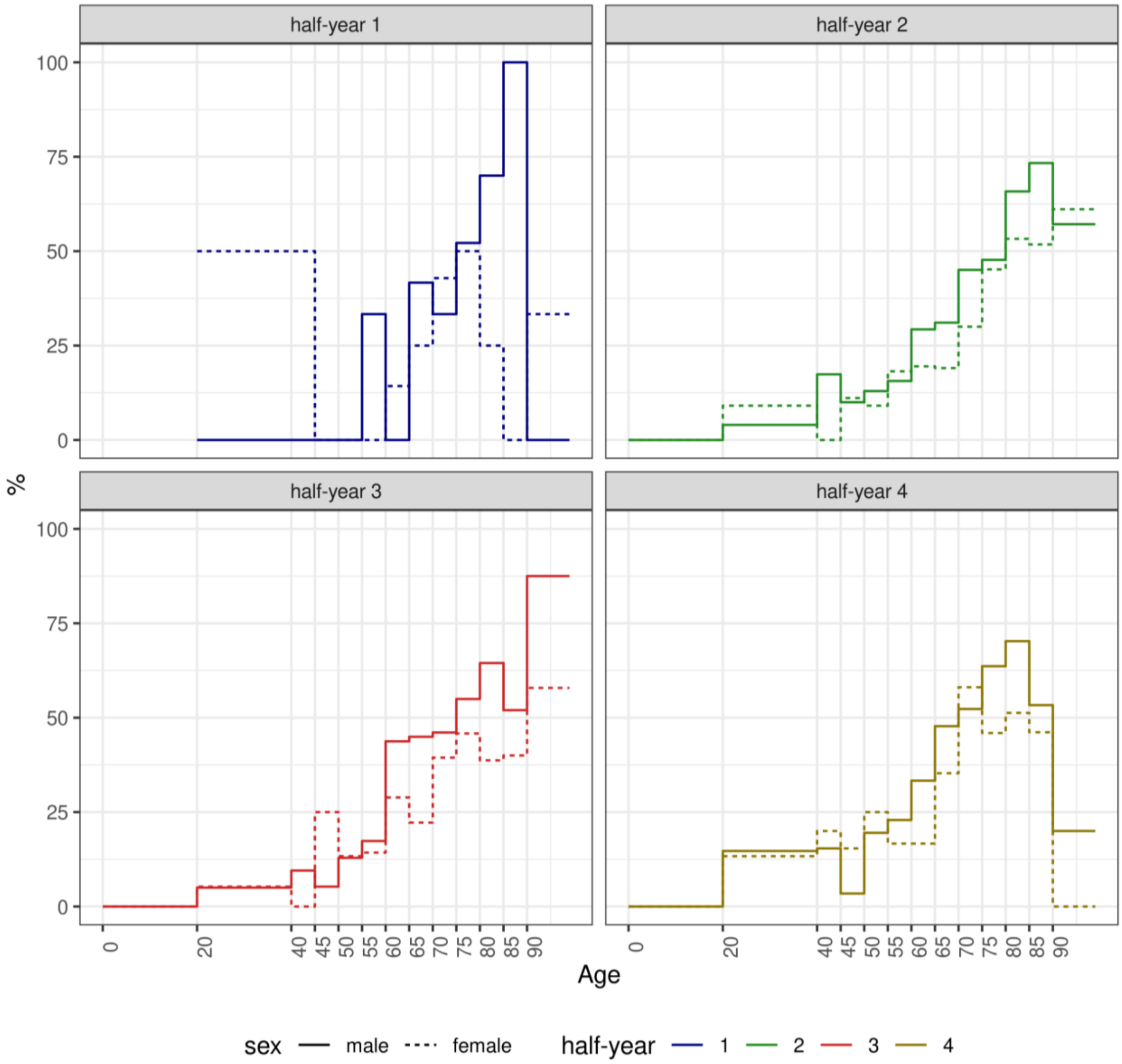

Table S 5 Multivariable logistic regression analysis in all patients with COVID-19 admitted to Austrian hospitals in 2020 and 2021 (n=68 193) with primary admission to an intermediate care unit (n=3 529) as the dependent variable. CI = confidence interval, OR = odds ratio.

Variable		OR	95% CI		*p*
Sex					
	Female	1.00			
	Male	1.46	1.36	1.57	<0.001
Age category [years]					
	19 and below	0.38	0.28	0.53	<0.001
	20 – 39	0.44	0.36	0.53	<0.001
	40 – 44	0.54	0.42	0.70	<0.001
	45 – 49	0.63	0.51	0.77	<0.001
	50 – 54	0.78	0.66	0.93	0.004
	55 – 59	0.89	0.76	1.04	0.14
	60 – 64	1.00			
	65 – 69	1.07	0.92	1.24	0.40
	70 – 74	1.13	0.98	1.31	0.09
	75 – 79	0.97	0.84	1.11	0.64
	80 – 84	0.79	0.68	0.91	0.001
	85 – 89	0.44	0.36	0.53	<0.001
	90 and above	0.24	0.18	0.31	<0.001
Half-year					
	Half-year 1	0.91	0.76	1.08	0.27
	Half-year 2	1.00			
	Half-year 3	1.04	0.96	1.13	0.35
	Half-year 4	0.77	0.70	0.85	<0.001
Region					
	East	1.00			
	North	0.15	0.06	0.39	<0.001
	South	0.22	0.09	0.58	0.002
	West	0.32	0.11	0.90	0.03

Table S 6 Multivariable logistic regression analysis in all patients with COVID-19 admitted to Austrian hospitals in 2020 and 2021 (*n*=68 193) with primary admission to an intensive care unit (*n*=8 304) as the dependent variable including an interaction term between sex and age category. CI = confidence interval, OR = odds ratio.

Variable	OR	95% CI		*p*
Sex				
Female	1.00			
Male	1.52	1.31	1.77	<0.001
Age category [years]				
up to 19	0.35	0.26	0.48	<0.001
20 – 39	0.34	0.28	0.42	<0.001
40 – 44	0.55	0.42	0.72	<0.001
45 – 49	0.55	0.44	0.70	<0.001
50 – 54	0.73	0.59	0.89	<0.001
55 – 59	0.88	0.73	1.05	0.16
60 – 64	1.00			
65 – 69	1.15	0.98	1.36	0.09
70 – 74	1.04	0.88	1.22	0.64
75 – 79	0.79	0.67	0.93	<0.001
80 – 84	0.51	0.44	0.61	<0.001
85 – 89	0.26	0.21	0.31	<0.001
90 and above	0.11	0.08	0.14	<0.001
Interaction: Sex × Age category				
sex × up to 19	0.84	0.56	1.27	0.41
sex × 20-39	1.34	1.04	1.73	0.02
sex × 40-44	1.10	0.80	1.53	0.56
sex × 45-49	1.26	0.95	1.67	0.11
sex × 50-54	1.09	0.86	1.39	0.46
sex × 55-59	1.05	0.85	1.31	0.64
sex × 60-64				
sex × 65-69	0.98	0.80	1.20	0.85
sex × 70-74	0.98	0.80	1.19	0.85
sex × 75-79	0.92	0.76	1.13	0.43
sex × 80-84	1.14	0.93	1.40	0.22
sex × 85-89	0.94	0.71	1.25	0.68
sex × 90 and above	0.91	0.58	1.43	0.69
Half-year				
Half-year 1	1.38	1.24	1.54	<0.001
Half-year 2	1.00			
Half-year 3	1.25	1.18	1.33	<0.001
Half-year 4	1.25	1.17	1.32	<0.001
Region				
East	1.00			
North	1.33	0.97	1.82	0.08
South	1.78	1.30	2.44	<0.001
West	1.48	1.05	2.10	0.03

Table S 7 Multivariable logistic regression analysis in all patients with COVID-19 admitted to Austrian hospitals in 2020 and 2021 (*n*=68 193) with primary admission to an intermediate care unit (*n*=3 529) as the dependent variable including an interaction term between sex and age category. CI = confidence interval, OR = odds ratio.

Variable	OR	95% CI		*p*
Sex				
Female	1.00			
Male	1.47	1.17	1.85	<0.001
Age category [years]				
up to 19	0.41	0.25	0.66	<0.001
20 – 39	0.32	0.23	0.44	<0.001
40 – 44	0.46	0.29	0.72	<0.001
45 – 49	0.57	0.40	0.83	<0.001
50 – 54	0.83	0.62	1.12	0.23
55 – 59	0.75	0.56	0.99	0.05
60 – 64	1.00			
65 – 69	1.10	0.85	1.42	0.46
70 – 74	1.14	0.89	1.45	0.30
75 – 79	1.05	0.83	1.33	0.66
80 – 84	0.87	0.69	1.10	0.26
85 – 89	0.45	0.34	0.60	<0.001
90 and above	0.24	0.17	0.35	<0.001
Interaction: Sex × Age category				
sex × up to 19	0.91	0.48	1.72	0.78
sex × 20-39	1.74	1.17	2.59	0.01
sex × 40-44	1.29	0.75	2.24	0.36
sex × 45-49	1.15	0.74	1.78	0.54
sex × 50-54	0.90	0.63	1.30	0.59
sex × 55-59	1.28	0.91	1.80	0.16
sex × 60-64	1.00			
sex × 65-69	0.95	0.70	1.30	0.75
sex × 70-74	0.99	0.74	1.34	0.97
sex × 75-79	0.87	0.65	1.16	0.33
sex × 80-84	0.82	0.61	1.11	0.20
sex × 85-89	0.95	0.65	1.38	0.79
sex × 90 and above	0.94	0.56	1.58	0.81
Half-year				
Half-year 1	0.91	0.76	1.08	0.26
Half-year 2	1.00			
Half-year 3	1.04	0.96	1.13	0.37
Half-year 4	0.77	0.70	0.85	<0.001
Region				
East	1.00			
North	0.15	0.06	0.39	<0.001
South	0.22	0.09	0.58	<0.001
West	0.32	0.11	0.89	0.03
